# The role of traditional medicine practice in primary health care within Aboriginal Australia: a review of the literature

**DOI:** 10.1186/1746-4269-9-46

**Published:** 2013-07-02

**Authors:** Stefanie J Oliver

**Affiliations:** 1 Kimberley Aboriginal Medical Services Council Inc, Dora St, Broome 6725, Australia

**Keywords:** Australian Aboriginal Health, Bush Medicine, Traditional Healers, Traditional Aboriginal and/or Torres Strait Islander Medicine, Australian Ethnomedicine

## Abstract

The practice of traditional Aboriginal medicine within Australia is at risk of being lost due to the impact of colonisation. Displacement of people from traditional lands as well as changes in family structures affecting passing on of cultural knowledge are two major examples of this impact. Prior to colonisation traditional forms of healing, such as the use of traditional healers, healing songs and bush medicines were the only source of primary health care. It is unclear to what extent traditional medical practice remains in Australia in 2013 within the primary health care setting, and how this practice sits alongside the current biomedical health care model. An extensive literature search was performed from a wide range of literature sources in attempt to identify and examine both qualitatively and quantitatively traditional medicine practices within Aboriginal Australia today. Whilst there is a lack of academic literature and research on this subject the literature found suggests that traditional medicine practice in Aboriginal Australia still remains and the extent to which it is practiced varies widely amongst communities across Australia. This variation was found to depend on association with culture and beliefs about disease causation, type of illness presenting, success of biomedical treatment, and accessibility to traditional healers and bush medicines. Traditional medicine practices were found to be used sequentially, compartmentally and concurrently with biomedical healthcare. Understanding more clearly the role of traditional medicine practice, as well as looking to improve and support integrative and governance models for traditional medicine practice, could have a positive impact on primary health care outcomes for Aboriginal Australia.

## Background

Traditional medicine practice (TMP) within Aboriginal Australia encompasses a holistic worldview which reflects that of the World Health Organizations definition of health, which is one of ‘physical, mental and social wellbeing and not merely the absence of disease or infirmity’ [1]. This worldview recognises good health as a complex system involving interconnectedness with the land, recognition of spirit and ancestry, and social, mental, physical and emotional wellbeing both of the individual and the community [2]. Indigenous Australians view ill health as the result of one of three causes – a natural physical cause, a spirit causing harm, or sickness due to sorcery [3]. The impact of colonisation and the subsequent displacement and disconnection of people both from their traditional lands and later from their traditional families has been significant in its subsequent effect in the use of traditional practices including traditional medicine [4].

The Alma-Ata declaration on primary health care (PHC) by the World Health Organization (WHO) in 1978 witnessed a response from several countries to improve their traditional medicine use and regulation of use within the primary health care model. PHC for Aboriginal and Torres Strait Islander Australians is currently addressed by either government controlled health services or community controlled health services (ACCHS) that offer biomedical health care and employment to trained Aboriginal Health Workers (AHWs). ACCHS are initiated and governed by the local Aboriginal Community to enable delivery of holistic and culturally appropriate healthcare to the respective community/ies [5]. This holistic approach in the evolvement from primary medical care to primary health care as adopted by the Alma-Ata declaration in 1978 has been praised, however there has been no mention of the incorporation of traditional medicine use within the design of these health services as other countries have [6]. It is acknowledged that in remote areas in other countries it is common for traditional medicine to coexist with biomedical healthcare as part of a pluralistic medical system [7]. It is unclear if this also applies to Aboriginal Australia and if so, to what extent traditional medicine is practiced and how it sits with the use of biomedical healthcare.

The purpose of this review is to identify available literature that examines or discusses the role (as in position/function) of TMP in a PHC setting within Aboriginal Australia. Treatment modalities within TMP for the review will be inclusive of Traditional Healers (TH), herbal medicines, ceremonies and healing songs [8]. Whilst it is recognised that bush foods also play a role in traditional health practices, specific articles on bush tucker and nutrition will not be included due to the limitations on the length of this review. TMP will be examined overall for the extent/level of use, *when* they are used (i.e. first or second line), *how* they are used (i.e. alone or in combination with biomedicine), *what* they are used for (i.e. types of illnesses) and the reasons behind the when, how and what.

## Methodology

Database searches were performed via the internet using Google, Google scholar, PubMed, Indigenous health info net, snowballing (reference citation), related government and non-government websites. Keywords used in the search were: Traditional/ Indigenous/ Aboriginal/ Torres Strait Islander/ bush/plant medicine; traditional medicine practices; ethnomedicine; traditional healer/practitioner; traditional health practices; AND one or more of the terms: primary health care; role of; integration; Australia; Aboriginal Australia/n. State library resources were also identified.

Literature found was inclusive for both qualitative and quantitative research articles and non-research articles such as letters/articles within journals or magazines and recorded audio interviews for urban, rural, remote and very remote Aboriginal Australia. Pharmacopoeias were excluded.

Literature included in the review either;

i. Examined/evaluated TMP amongst Aboriginal Australians, including the reasons surrounding using or not using TMP, where TMP included traditional healers, herbal medicine, ceremony, healing songs.

ii. Documented any TMP amongst communities including the type of ailments treated.

iii. Discussed/researched TMP as PHC.

iv. Discussed/researched TMP alongside/with biomedicine and biomedical healthcare services.

Literature excluded from the review either;

i. Focussed on documentation or identification of medicinal plants and their preparation methods and/or uses.

ii. Examined the biological activity or phytochemical constituents of medicinal plants identified;

iii. Examined traditional midwifery.

iv. Discussed or reviewed TM methods and philosophy without examining any role within PHC.

v. TMP was not at the primary health care level.

vi. Examined non-Indigenous Australian models of TM (i.e. Traditional Chinese Medicine) within Aboriginal and Torres Strait Islander communities.

The total number of articles found that met inclusion criteria was 13, dating from 1992–2013. 12 of these were reviewed in detail and cited throughout the review (one article was a research proposal). No qualitative tools such as MOOSE were used. The review was conducted solely by the publishing author.

## Key findings

The review is themed according to the setting of PHC. The first group is PHC based at an established health clinic with two sub-groups – clinics offering any aspect of TMP alone or in combination with biomedical health care, and clinics offering only biomedical health care. The second group is household/community PHC also in two sub-groups - TMP offered by THs, and TMP offered by other community members e.g. family.

### Established primary health care clinics

#### ***Clinics offering TMP alone or in combination with biomedicine***

The Office of Aboriginal and Torres Strait Islander Health (OATSIH) report on provision of health-care services in 2010–2011 includes traditional healing and bush medicine service provision [9]. Questionnaires were distributed to all participating Aboriginal and Torres Strait Islander biomedical health services that receive funding from OATSIH for provision of primary health care. Nearly 100% of services responded. The results showed that in the year 2010–2011 the percentage of health clinics that offered services of traditional healers was 19.7% and that of bush medicine 12.4%. This compares with previous years – 2009–2010 at 14.8% and 9.9% and 2008–2009 at 17.9% and 10% respectively, showing a slight increase in the 2010–2011 periods. Therefore statistically within government funded established primary health care clinics in Aboriginal Australia roughly one fifth offer traditional healers and one tenth offer bush medicines as part of the healthcare service. There is however a lack of detail within the report surrounding this service provision. Details such as how often these services were provided, when, why and how they were provided with respect to biomedical healthcare and if these service provisions resulted in employment within the health service were not reported. Clearly more descriptive analysis is required to gain better understanding of TMP in PHC.

Apart from the OATSIH report, no formal studies were found for the use of TMP within PHC clinics in Australia. However there were written anecdotal reports from Aboriginal health workers and nurses employed within select health clinics for the storage and use of bush medicines, and sometimes THs, within the clinic [10,11]. These anecdotal accounts of TM use were all based in the Northern Territory and describe that bush medicine plays a role in the ‘treatment of medical, surgical and spiritual ailments’ [10], for ‘infected sores and scabies’ [12], that ‘bush medicines were kept at the back of the clinic’ [11] and that bush medicines plants have been established in the front of the health centre and that ‘soon we (Yolngu) will be using it with the western medicines’…‘working together with ‘balanda” (non-Aboriginal people) [13]. Observation of a TH visiting to the clinic (i.e. not resident) to heal a girl successfully with fits was also made by one author [11]. These anecdotal accounts give us little information regarding the extent of use or the reasons for use of TM, and are unreliable as sources of current practice as all three accounts were written 9 years or more ago.

The Akeyulerre Healing Centre in Alice Springs offers stand-alone TMP (THs and bush medicines) in a culturally safe place where traditional knowledge and practices can be shared and practiced. An Australian Broadcasting Network (ABC) interview conducted with an ethnobotanist researching the use of bush medicines and a local elder women discussed the use of specific bush medicines made by local community people provided at the centre [14]. It was explained that many people in the local community, as well as visitors from other communities, come to get the bush medicines for a range of ailments such as colds and flus, sore muscles, wounds, headaches and skin rashes, and that the elders are passing on this knowledge to young people, and that (bush medicines’ have ‘always been used’ While this interview may tell us that bush medicines are being widely used, qualitative and quantitative data was not investigated to understand the ‘why’, ‘how’, and ‘when’ of TMP.

Similar to Alice Springs local elder women in the Western Australian Kutjungka community Balgo Hills (Wirrimanu) have formed the Palyalatju Maparna Health Committee which provides bush medicines to the local biomedical health clinic at Balgo, the local community and surrounding communities [15]. The elder woman who was interviewed for Indigenous newslines, describes that

‘*the beauty of bush medicine is it makes us feel good*, *and it feels good using our own ways to make community strong*’, and

‘*blackfellas and whitefellas come and tell us*, ‘*I*’*m feeling better from your bush medicine*, *can I get some more*?’

The article describes that bush medicine is used on its own or in combination with modern medicine and types of ailments that it is used for include headaches, diabetes and wounds to name but a few. In April 2011 the funding was ceased and the committee dissolved [16]^a^. The incorporation of the Palyalatju Maparna Health Committee could be seen to play an important role in the community for access to bush medicines for primary health care. Whilst further research is justified in assessing this role both qualitatively and quantitatively the article does give us an indication that the provision of bush medicines by local women elders improved TMP for the Balgo community.

Not yet published a pilot study is under way to assess the combination of use of THs and biomedicine with a two-phase qualitative project in Queensland, after studies of Diabetes Type 2 in four regional/rural Aboriginal communities found that a number of patients still utilised traditional healing practices [17].

#### ***Clinics offering only biomedicine***

A qualitative survey by way of a questionnaire was developed in Aurukun Health clinic, Cape York Peninsula in far North Queensland, to determine the extent of use of bush medicines by clients of the health service and for what types of illnesses medicines were used for [18]. Permission for the survey was gained from the Queensland Health Ethics committee and the survey was conducted and filled in by clinic staff due to low literacy levels of clients. As a consequence the survey did not go well and no understanding of bush medicine use was gained as a result. The set up of this survey could be seen to fail on several levels – identification of some of these reasons has been made by reviewers of the research project [18]. Cross-cultural communication, cultural sensitivities for sharing of knowledge and re-enforcing of negative colonialist experiences through the research process were reasons identified. This highlights difficulties in qualitative field research, and the need for sound cultural understanding and putting time into the design of research and building trust relationships with community before attempting research.

### Primary health care in the household or community

#### ***Traditional healers (THs)***

The Ngaanyatjarra Pitjantjatjara Yankunytjatjara (NPY) Women’s Council Aboriginal Corporation employs traditional healers as part of the services offered by the organisation. The ‘Ngangkari program’ offers health and mental health outreach services within NPY lands which covers about 25 communities in the tri-state area of NT, SA and WA [19]. Both a book has been published about these traditional healers, or Ngangkari [20], and an interview was recorded on ABC which examined the role of the THs [19]. It is reported that Ngangkari work hand in hand with the mainstream health services both in primary and tertiary health care and are recognised by the mainstream medical doctors, working alongside and in co-operation with them. From the report of one TH, the role of the TH is in combination with biomedical care;

“*Often a doctor will say*, ‘*listen*, *we*’*ve got this sick child here*, *could you give this child a treatment and after you*’*ve done a treatment we*’*ll give it appropriate medicine*’…*there*’*s a lot of cooperation these days and respect*.”

The sequence of these events is noted. First line, the patient is seeing the ‘western’ doctor. This doctor then refers to the TH for a treatment who then refers back to the western doctor for pharmaceutical medicine (rather than traditional herbal medicine). No information is given surrounding this process that informs the reader of the extent of this practice, such as was it the western doctor who felt that the patient would benefit from the TH, is this process used on every patient or was it at the patients request? What we can determine from this account is that there is mutual respect between the western doctor and TH in this situation. As one TH states [20];

‘..*today it is recognised that a ngangkari is a doctor too*. *Doctor*, *ngangkari*. *Ngangkari*, *doctor*. *Same thing*.’

An ethnographic account of THs in the Kutjungka region of North Western Australia by observation of artistic description of healing practices was made by McCoy [21]. The account sought to understand by way of this observation as well as conversation with community members about health behaviour (after their permission was sought). The THs of this region are known as ‘Maparn’, that is ‘men or women who respond to people’s sickness using a traditionalist model of diagnosis and healing’. Observational reports stated that many people visit the Maparn first, especially if they consider their sickness to be serious, and that sometimes Maparn will visit the clinic, especially if a family member requests their presence. An account of a young man in his twenties who used services of both the Maparn and the health clinic concurrently was described – the young man would visit the Maparn in the morning and the clinic in the afternoon. The availability of Maparn may affect the role that TM plays – in some communities Maparn have passed on and in others they have given up their practice, which means that Maparn from other communities will need to travel. Although this type of research provides detailed and accurate description, it does lack objectivity and does not give us a reliable indication to the extent that Maparn are incorporated in health behaviour of the community, for example a percentage of community members that use Maparn, and if this use is associated with cultural affiliation.

More ethnographic research was completed within the Warlpiri community in the Northern Territory, specifically on two recorded illness episodes to examine health behaviour of people using ngangkari’ [22]. In his observations the author discovered that the use of bush medicine was used to treat specific symptoms of illnesses and included coughs, colds, wounds and sores, and that every adult and many children had some knowledge of bush medicine. If the disease however was caused by sorcery then an Ngangkari was consulted. Two illness-related cases were followed to examine health behaviour. The first case was a 44yr old male who consulted several Ngangkari over a period of weeks before finally visiting the clinic (biomedical) after his condition was not improving and becoming worse. The second case was a 33yr old girl who after years of biomedical healthcare ceased visiting the clinic (except to collect her long-term medicines) to engage with an Ngangkari. These two cases give an example of different age and gender who both utilised THs in different sequences, and whilst the same subjectivity may apply as for the above ethnographic study and lack of understanding of the level of the community who engage with Ngangkari, it does give us an indication of the role of the TH based on health beliefs of illness causes.

#### ***Household/family members***

Reasons for use of bush medicine amongst cancer patients or their family members were recorded in a study based in Western Australia [23]. The qualitative analysis was by way of individual in-depth interviews, observations and field notes. Results were analysed thematically into reasons why or why not bush medicine was used demonstrating both the role and use of TM. Consent was given from the Aboriginal reference group involved and this group was consulted throughout the study period. Thirty seven in-depth open-ended interviews were conducted in English, including one rural and two remote participants whilst the remainder resided in urban Perth, Western Australia. Out of these 11 types of cancer were identified and only 11 of the 37 interviews were used as the focus for the paper. The results of the study found that bush medicine played a role in symptom relief from chemotherapy or stress associated with the situation. In some cases people chose TM over western medicines and vice versa depending on their situation and beliefs surrounding chemotherapy and TM. Such situations were likely to be concern over leaving family to come for chemotherapy treatment, adverse reactions from chemotherapy, limited access and knowledge of bush medicines, and uncertainty about bush medicine interactions with cancer medicine [23]. As one participant reported

‘*I tried* [*bush medicine*], *but*, *yeah*, *I think it reacts with all my tablets I*’*m taking*.’

This study gives us a valid indication that TM plays an important role in cancer and its use depends on cultural knowledge, access to TM, concerns about integrative healthcare, and location, however a bigger sample size would have given this study more reliability.

## Discussion

It is evident that there is a gap in literature that seeks to examine specifically the role of TMP for PHC rather than the philosophy or description of Aboriginal TM. Although evidence exists for the use of TMP in primary health care, either alone or in combination with biomedicine, reliable and valid research is lacking. Specifically, there is a paucity of literature that seeks to examine the role of traditional treatment modalities of ceremony and healing songs, instead the focus is on traditional healers or bush medicines. Saying this, the literature found does give us an indication that TMP exists and this enables a discussion about its role in PHC.

### Examining the role of TMP

The role of TMP can be analysed quantitatively and qualitatively. Quantitatively the OATSIH report is the best example. The percentages of overall service provision serves as a useful tool to examine the extent of TMP. Combining both THs and bush medicine gives us a figure of 32.1% of Aboriginal primary health care services across Australia that offered some form of traditional medicine practice in the year 2010–2011 (excluding bush tucker programmes), that is 76 out of 236 services [9]. Considering that prior to colonisation 100% of primary health care would have been with TM, this tells us that the current extent of TMP is relatively low. Quantitatively this report gives us no indication for reasons and extent of use of these services within an individual clinic, such as how often or what type of illness. More questions need to be designed into the report if these reasons are to be identified and examined.

Qualitatively, the role of TMP can be described as sequential (i.e. in sequence), compartmental (i.e. treatment chosen is based on illness type) or concurrent (i.e. at the same time) [24]. The ethnographic research conducted [21,22] show that people within the relevant communities studied exhibit all 3 types of health behaviour for using THs. The understanding of disease cause was identified by both these authors to underpin the choice of treatment, that is the ‘why and what’ of TM use. Desert people believed that ‘new’ diseases such as diabetes and cancer lay outside the powers of the THs, whereas illnesses believed to be caused by spirit resulted in people seeking traditional healing methods [21]. This was reinforced by observations from the second author that illnesses thought to result from contact and colonisation were considered ‘white’ illnesses including diabetes and that ngangkari cannot help [22]. These types of health behaviour were not limited to the household/community. THs were reported to visit the clinic at request [10,11,21], however it is unclear from these reports how this fits in to the sequential/concurrent or compartmental behaviour model. The ngangkari account from the NPY women’s council described in the review exhibits both sequential and concurrent health behaviour of a client. This behaviour could be affected by the residency or employment status of the TH within the health services. The NPY Women’s council state clearly that THs are employed for health services, whilst other accounts do not mention employment status. It is reported that THs were employed in Australia by the Northern Territory Department of Health in the early 1970s, however a training course to teach traditional healers about western medical practices was soon replaced by the training program for AHWs [24]. Despite this, Trudgen [25] describes (2000) that he does not know of one *marrŋgitj* (traditional doctor) employed in a health clinic, knows only one herbalist who is employed as a cleaner and one AHW who has learned both traditional healing and western medicine in Arnhem land.

### Medical pluralism

Medical pluralism is essentially the adoption and integration of biomedical healthcare with TMP, or ‘concurrent’ treatment as described above. With reference to integration, Chan in her speech at the WHO Congress on Traditional Medicine [26] comments that;

‘*The two systems of traditional and western medicine need not clash*. *Within the context of primary health care*, *they can blend together in a beneficial harmony*, *using the best features of each system*, *and compensating for certain weaknesses in each*.’

Concern over interactions between pharmaceutical medicines and bush medicines was identified within the study on cancer and bush medicine as a reason for not wanting to use bush medicines [23]. While not articulated in any of the research, the area of uncertainty for drug-plant interactions should be considered from the other perspective also – that is non-compliance of pharmaceutical medicine due to a desire to use bush medicine and not wanting to mix the two.

In the 1970s the concept of ‘two ways’ was introduced in the Northern Territory of Australia, incorporating both traditional healthcare and biomedical healthcare, however was dismissed by the late 1990s for reasons unknown [22]. Ivanitz [27] also reports in 1999 that individuals will often visit both a traditional healer and the biomedical health clinic, which is confirmed in more recent times by the observational and anecdotal accounts of Mccoy [21], Saethre [22] and the NPY Women’s Council [20]. As one Yolngu member puts it [28];

‘*when we put* [*western medicine and traditional Yolngu healing*] *together*, *we strong* – *both feet strong*. *We can see with a clear mind*. *Stand strong together*.’

From these accounts, integration can be viewed as not only the combination of pharmaceutical and plant medicine but also the combination of traditional healers and western medical doctors. Integration of both systems requires an understanding of the social and cultural constructions of each medical system and the complexity of the whole.

### Association with culture

The association or lack of association with culture was shown to underpin the choice of using TM in the study on cancer patients and their use of bush medicines in Western Australia [23], where one participant reports that

‘*we didn*’*t use traditional medicine or anything like that*. *Because we are not traditional Aboriginal*, *and our family was Christian based*, *and so*…*We put our trust on God*.’

It is clear from the interviews with elders from both Balgo [15] and the Akyulerre Healing centre [14] that they believe using TM keeps culture strong. As is described in one article [12];

‘*using bush medicine*…*raises the self*-*esteem and makes aboriginal people more self*-*reliant since they all have access to the trees*.’

On the flipside, a lack of understanding about social constructions of western medical systems and associated culture by Aboriginal and Torres Strait Islander peoples who are traditionally oriented, could mean that there is a perceived failure of biomedical treatment. A perceived failure of treatment would then impact on the role and health-seeking behaviour of people, especially for illnesses where pharmaceutical medicine is being used to treat in a preventative role, such as the prevention of micro- and macro-vascular complications of diabetes type 2.

Another influence that has been identified in the above review is that of gender. The Maparn THs in the Kutjungka were reported to be generally male, although there are some female Maparn. Conversely, it is traditional for the women and children to collect and prepare bush medicines and become the ‘household healer’ [21]. A study performed in the Amazon in Brazil [29] researched the different roles of gender in TM and highlighted power struggles within biomedical system for those women who had traditional plant medicine knowledge and known as the ‘household healers’ who felt disempowered by the system. The resultant effect was for these women to not access the biomedical healthcare and treat their children at home with TM. This highlights the importance to incorporate gender roles within research for TMP.

### Sharing of knowledge

It is acknowledged that a limitation of this review is the lack of written documentation for TM as traditional documentation of medicinal plants is by way of paintings [30] and passing down of knowledge through generations by story and songs. Another limitation is the reluctance to share knowledge with outsiders. This may be due to cultural reasons or mistrust regarding the way that this information will be used. A lack of building appropriate trust relationships and respect for the worldview of Aboriginal people from researchers con contribute to the potential for an unwillingness to disclose knowledge. The authors of the questionnaire survey at the Aurukun Health Clinic also came across difficulties for sharing of knowledge. Reasons cited were that bush medicine is ‘secret business’, that respondents thought that it might offend clinic staff if they knew they were also using ‘*opar*’ (bush medicine) [18]. Within the study of Aboriginal people and cancer one patient did not want to share information on the bush medicine used, highlighting ‘the tension between what is allowed to be public knowledge by Aboriginal people and what remains private.’ [23]. The WHO Traditional Medicine Strategy [31] outlines that protection of this knowledge is important and needs to be considered as a different system than the current intellectual property rights agreement. The strategy clearly states that traditional knowledge is ‘owned by the community and is to be used for its benefit’. Permission therefore to document and use this knowledge must be sought in a way that is reciprocal with and reflective of the will of the community.

### Governance

It is one thing for TM to be practiced in traditional ways at a local level and another for it to be recognised as part of a national healthcare strategy. In Australia there are no national government organisations for Aboriginal TMP. There is one national non-government organisation currently in operation. The recently formed Indigenous NGO Aboriginal and Torres Strait Islander Healing Foundation Ltd [32] have supported new projects for Indigenous healing such as the ‘Angangkere Healing Project’, which is for THs and being run out of the Akyulerre healing centre in Alice Springs, and the ‘Rumbulara Traditional Healing Centre’ in Victoria run under as part of the Rumbulara Aboriginal Co-operative Ltd which will be established separate to the current medical service PHC clinic. In contrast New Zealand hosts a National Board of Maori Traditional Healers and in 1999 the Ministry of Health published a set of standards for traditional Maori healing [33], whilst in the US in 2002 the previously formed Association of American Indian Physicians approved a resolution acknowledging and supporting Native American traditional healing and medicine as part of the spectrum of health care appropriate for Native Americans [34]. The latest response to the development of a National Aboriginal and Torres Strait Islander health plan was for an increased recognition and inclusion of Aboriginal traditional medicine within the health plan [35]. Ivanitz [27] also recommends that ‘policies need to be developed and implemented that ....address the use of traditional medicine alongside mainstream medicine’. Regulated governance structures can potentially improve the quality of TMP including the reduction or removal of quackery by practitioners.

## Conclusions

It is evident that good research design that takes into account Aboriginal worldviews, reciprocity and cultural sensitivities is paramount to reliable outcomes for research within this field, and there are very few well-designed research articles examining the role of TMP in PHC within Aboriginal Australia. Whilst there is a paucity of research identified, the existing literature identified establishes reasons underpinning the use of TM and when it is used, alone or combination with biomedicine. These reasons are identified as association with culture, access to bush medicines and THs and health beliefs about disease causation, such as using THs for perceived spiritual or sorcery causes of illness and bush medicines for symptom relief of physical causes of disease for a range of ailments including colds and flus, wounds, headaches, aching muscles and skin rashes. It is also clear that health seeking behaviour is complex and medical pluralism exists, and more focus on integration of TM with conventional medicine is warranted. It is clear that there is willingness amongst some communities to strengthen TMP and keep culture strong, however changes to and support for integrative and governance models for TMP need to be made and support increased to reduce the risk of the loss of knowledge as generations shift. Australia could benefit by looking to other nations to improve this support and strengthen governance for traditional Aboriginal medicine. Understanding more clearly the role of TMP by improving the quality and quantity of research within Aboriginal Australia could potentially improve primary health care outcomes for Aboriginal and Torres Strait Islander Australians.

## Endnote

^a^Since the completion of this article the author has discovered that the health committee has received funding for it to re-open.

## Abbreviations

PHC: Primary Health Care; NGO: Non-Governmental Organisation; TM: Traditional Medicine; TMP: Traditional Medicine Practice; WHO: World Health Organization; AHW/s: Aboriginal Health Worker/s; ACCHS: Aboriginal Community Controlled Health Services; TH/s: Traditional Healer/s; OATSIH: Office of Aboriginal and Torres Strait Islander Health.

## Competing interest

The first draft of this article was completed when SO, a registered pharmacist and herbalist, was self-employed. SO was subsequently employed as a pharmacist at the Kimberley Aboriginal Medical Services Council (KAMSC). This review was researched and written independent of employment and the author declares no financial assistance was given nor any financial gain is promised as a result of publication.

## Author’s contribution

SO has conceptualised, researched, designed and written the review as well as read and approved the final manuscript.
